# Supplementary material for: Stacking Model‐Based Classifiers for Dealing With Multiple Sets of Noisy Labels
Source: Biom J. 2025 Mar 12;67(2):e70042. doi: 10.1002/bimj.70042 (PMC11898607; doi:10.1002/bimj.70042)
Supplement: Supplementary file 1 — Supporting Information [file BIMJ-67-e70042-s001.zip › density-based-ensemble-model-main/competitors_real_data/netlab-master/netlab-master/help/rbfunpak.htm]

Netlab Reference Manual rbfunpak


# rbfunpak

## Purpose

Separates a vector of RBF weights into its components.

## Synopsis

```
net = rbfunpak(net, w)
```

## Description

`net = rbfunpak(net, w)` takes an RBF network data structure `net` and
a weight vector `w`, and returns a network data structure identical to
the input network, except that the centres
`c`, the widths `wi`, the second-layer
weight matrix `w2` and the second-layer bias vector `b2` have all
been set to the corresponding elements of `w`.

## See Also

`rbfpak`, `rbf`

---

**Pages:**
Index


---

Copyright (c) Ian T Nabney (1996-9)
